# Advances in malaria vaccine development: report from the 2017 malaria vaccine symposium

**DOI:** 10.1038/s41541-017-0035-3

**Published:** 2017-11-30

**Authors:** Camila Henriques Coelho, Justin Yai Alamou Doritchamou, Irfan Zaidi, Patrick E. Duffy

**Affiliations:** Laboratory of Malaria Immunology and Vaccinology, National Institute of Allergy and Infectious Diseases, National Institutes of Health, Bethesda, MD USA

## Abstract

The Malaria Vaccine Symposium occurred at Johns Hopkins University in Baltimore, MD, USA on April 25th, 2017, coinciding with World Malaria Day and the WHO announcement that the RTS,S malaria vaccine would begin pilot implementation programs in Ghana, Kenya, and Malawi in 2018. Scientists from several disciplines reported progress on an array of malaria vaccine concepts and product candidates, including pre-erythrocytic vaccines that prevent infection, blood-stage vaccines that limit infection and disease, and transmission-blocking vaccines that interrupt the spread of infection. Other speakers highlighted the immunological and genetic considerations that must be addressed by vaccinologists to yield the most efficacious vaccines. Here, we highlight the advances in malaria vaccinology that were reported at the symposium.

## Introduction

New strategies are urgently needed to stem the malaria tide that causes hundreds of millions of clinical cases and claims hundreds of thousands of lives each year.^1^ Although individual protective measures for mosquito control (bednets, insecticides, and repellants) are being prioritized in endemic areas, the elimination of the disease is unlikely to be achieved in many areas without a vaccine. Malaria vaccines have long been a research priority, but only this year the World Health Organization announced that a malaria vaccine would advance to implementation studies. To highlight progress and recent advances in this field, Prof. Fidel Zavala (Department of Molecular Microbiology and Immunology, Johns Hopkins Bloomberg School of Public Health) and Dr. Robert Seder (Chief of the Cellular Immunology Section in the Vaccine Research Center, National Institute of Allergy and Infectious Diseases (NIAID), National Institutes of Health), invited leading scientists around the world to share the most recent updates from their research at the Malaria Vaccine Symposium. In this meeting report, we highlight the progress on different malaria vaccine concepts (Table 1), as well as on immunologic and genetic findings that may impact malaria vaccine responses and efficacy.

**Table 1:** Current malaria vaccines under preclinical development or in clinical trials

Parasite stage	Vaccine classification	Current status
**Pre-erythrocytic stage**		
PfSPZ vaccine	Whole organism (radiation attenuation)	Phase II
GAP vaccines	Whole organism (genetic attenuation)	Phase I
RTS,S	Subunit	Phase IV
CVac	Whole organism (chemical attenuation)	Phase I
**Blood stage**		
Chemically attenuated parasites	Whole organism	Preclinical
AMA1-RON2	Subunit	Preclinical
PfRH5	Subunit	Phase I
**Mosquito stage (TBVs)**		
Pfs25	Subunit	Phase I
Pfs230	Subunit	Phase I
Pfs47	Subunit	Preclinical

Pre-erythrocytic, blood-stage and transmission-blocking vaccines are being evaluated in clinical trials (denoted as phases I to IV) or are being tested in rodent or non-human primate models (preclinical status).

## Pre-erythrocytic vaccines

Pre-erythrocytic vaccines target the clinically silent sporozoite and liver stages of *Plasmodium*. These vaccines aim to eliminate parasites during early infection and, if highly efficacious (i.e., induce sterilizing immunity), will avert disease and interrupt transmission. Session chair Fidel Zavala noted that whole sporozoite vaccines were shown to protect humans from *Plasmodium falciparum* in the 1970s,^2^ and current whole-organism vaccine approaches include irradiated parasites, genetically modified parasites, and infection in conjunction with chemoprophylaxis (called chemoprophylaxis vaccination or CVac). Recent progress in manufacturing whole organism vaccines has prompted several vaccinology questions: (1) Can whole sporozoite vaccines be improved? (2) What are the optimal doses, routes of administration, and adjuvants that confer sterilizing immunity? (3) Can other antigens be included? (4) Are we exploring all protective immune mechanisms? (5) Does efficacy differ in populations from endemic versus non-endemic areas? (6) What is the impact of antigen polymorphisms?

Dr. Robert Seder, from the NIAID Vaccine Research Center, shared results from trials of the PfSPZ vaccine, manufactured by Sanaria Inc. PfSPZ vaccine induces a breadth of responses that includes CD4, CD8, and gamma delta T cells, as well as antibody responses. PfSPZ vaccine also induces antigenic breadth. PfSPZ vaccine efficacy in malaria-naïve adults depends on direct venous inoculation to induce tissue resident T cells in the liver, and the dose determines the degree and durability of homologous and heterologous protection.^3–6^ Trials of PfSPZ vaccine in Mali confirm efficacy against naturally transmitted parasites, and studies in other African sites are underway to evaluate its use as a tool for malaria eradication.^7^ In Kenya, PfSPZ vaccine is currently being tested in 5–12-month-old infants for safety and efficacy against malaria infection. Other ongoing investigations of PfSPZ vaccine seek to optimize the dose/regimen, incorporate additional vaccine strains, and compare efficacies versus chemoprophylaxis vaccination and genetically attenuated parasite (GAP) vaccines.

Dr. Stefan Kappe, the Director of Translational Science at the Center for Infectious Disease Research in Seattle, reported on the development of GAP vaccines. Deletion of the pre-erythrocytic stage-expressed genes SAP1, P52, and P36 arrests parasite growth in hepatocytes at the early liver stage. Vaccination with triple gene knockout *P*. *yoelii* parasites conferred sterilizing immunity to mice.^8^ Furthermore, sera from human subjects immunized with the *P*. *falciparum* triple gene knockout GAP vaccine,^9^ when passively transferred into humanized-liver mice, protected against infectious *P*. *falciparum* sporozoite challenge, indicating that antibodies contribute to GAP vaccine immunity.

This *P*. *falciparum* early liver stage-arresting, triple gene knockout GAP vaccine has been shown to be safe in human subjects when delivered by mosquito bite.^8^ In partnership with Sanaria, GMP manufacturing of cryovialed *P*. *falciparum* GAP vaccine is planned, and will support trials using direct venous inoculation in the USA and Africa. While this first generation GAP vaccine moves through clinical development, it is thought that the optimal GAP vaccine might allow parasite development through late liver stage schizogony with subsequent developmental arrest before formation of infectious exo-erythroctyic merozoites. This type of late liver stage-arresting GAP vaccine candidate has been shown to induce superior protection against sporozoite challenge as well as stage-transcending protection against a rodent malaria blood-stage challenge in mice, both mediated by antibodies and T cells.^10^ A *P*. *falciparum* late liver stage-arresting GAP vaccine has yet to be generated and is a central focus of GAP vaccine research.

Dr. Robert Sauerwein from Radboud University, Netherlands reported progress with chemoprophylaxis vaccination, or CVac. In previous studies, CVac using sporozoites delivered by mosquitoes induced both polyfunctional T cells and antibodies, and conferred long-lasting protection against controlled human malaria infection (CHMI) with homologous parasites.^11^ Recently, CVac using cryovialed sporozoites also induced sterilizing immunity in malaria-naïve adults against homologous CHMI, even at accelerated vaccine schedules completed within a month.^12^ Future studies will explore CVac strategies that may confer long-lasting heterologous protection by incorporating different *P*. *falciparum* strains, which will be evaluated for their ability to confer protective efficacy in the field.

Dr. Ripley Ballou, the head of the GlaxoSmithKline (GSK) Global Vaccine Center arm, described the arc of development for the subunit malaria vaccine RTS,S that completed phase III trials in recent years. The RTS,S vaccine is composed of the repeat region of the circumsporozoite protein fused to the Hepatitis B virus surface antigen, and is adjuvanted with the proprietary AS01 adjuvant. Dr. Ballou summarized key studies demonstrating vaccine safety and efficacy in adults, children, and young infants in sub-Saharan Africa.^13,14^ In the phase III multicenter efficacy trial that enrolled 15,459 children in 11 centers across seven African countries, RTS,S/AS01 vaccination is estimated to have prevented 829 clinical malaria episodes per 1000 children over 18 months of study follow-up.^15^ Future studies will evaluate RTS,S integration into the EPI (expanded program of immunization) schedule. Private-public partnerships will be required to finance large scale phase IV vaccine trials to ensure long term success of future vaccines against malaria. For the pilot implementation studies in Ghana, Kenya, and Malawi, 750,000 children will be randomized to vaccinated and unvaccinated clusters, with four doses of RTS,S/AS01 administered in the vaccine testing group.

## Blood stage vaccines

To open the session, Dr. Carole Long from the NIAID reviewed the obstacles to blood stage vaccine development, including antigenic polymorphism of both merozoite as well as infected erythrocyte surface proteins, redundancy in the merozoite invasion pathways including both sialic acid-dependent and sialic acid-independent pathways,^16^ and difficulties expressing conformationally correct proteins. Studies thus far suggest that high antibody concentrations are needed to control merozoite invasion in vivo, and existing assays have failed to predict protection in humans. Trials of merozoite proteins have been disappointing, but ongoing efforts seek to improve the immunogenicity and functional activity of the antigens. New antigens as well as new antigen combinations might give additive or synergistic activity, and hence are a research priority for several groups.

Dr. Michael Good from Griffith University (Australia), shared recent findings from studies of chemically attenuated whole blood stage parasite vaccines. The chemical attenuation procedure developed by his laboratory entails incubation of blood stage parasites with a DNA-binding drug (Centanamycin or Tafuramycin-A), after which parasites are washed and resuspended for inoculation into mice.^17,18^ It is thought that these compounds affect parasite replication by irreversibly alkylating parasite DNA in polyA rich regions (reviewed in ref. ^19^). Immunization of mice with chemically attenuated blood-stage parasites, either as a single dose of *P*. *chabaudi* or three doses of *P*. *yoelii*, has induced homologous and heterologous immunity in a CD4 + T cell-dependent fashion.^17,18,20^ These vaccines activated peripheral blood CD8^+^ T cells but did not confer cross-stage protection, and blood-stage protection remained intact after CD8^+^ T cell depletion.^17^ Similar findings in *Aotus nancymae* monkeys^21^ prompted a pilot study in humans to assess safety and immunogenicity of *P*. *falciparum* blood-stage parasites chemically attenuated in vitro (reviewed in Stanisic and Good manuscript *in preparation*). Lysed infected erythrocytes do not induce protective immunity^18^ and the requirement for intact infected erythrocytes as an immunogen poses some challenges, such as the risk of induction of anti-erythrocyte antibodies and ethical issues regarding red blood cell products for some individuals. Novel approaches are under investigation in order to develop semi-synthetic blood-stage vaccines as alternative immunogens that can move forward into human studies.^22^

Dr. Simon Draper, from Oxford University (UK), described progress using the antigen *P*. *falciparum* RH5 to block merozoite invasion of erythrocytes and prevent blood-stage malaria infection. PfRH5 has been identified as the first highly conserved target from the merozoite to be susceptible to vaccine-induced broadly neutralizing antibody.^23^ In *Aotus* monkeys, PfRH5-based vaccines induced antibodies and conferred protection against a virulent heterologous *P*. *falciparum* challenge.^24^ Protection was associated with anti-PfRH5 antibody concentration and with in vitro parasite-neutralizing activity. More than 60% in vitro growth inhibition activity (GIA) was observed with the purified IgG, and the minimal serum concentration of anti-PfRH5 IgG required to protect an animal was defined as 200 µg/mL.^24^ A first generation PfRH5-based vaccine is being tested in clinical trials in Oxford and Tanzania; and studies to improve the vaccine include identification of critical epitopes in PfRH5,^25^ incorporation of PfRH5 immunogens into virus like particles, and manufacture of a clinical protein vaccine product in a *Drosophila* S2 cell line.^26^

Dr. Prakash Srinivasan from John Hopkins University (Baltimore, USA), described progress developing AMA1-RON2L complex as another vaccine that targets merozoite invasion. AMA1-only vaccines have failed to protect against homologous CHMI,^27^ despite field studies suggesting that AMA1 vaccines conferred activity against homologous parasites.^28^ AMA1 forms a complex with RON2 protein at the junction between merozoite and erythrocyte surfaces to initiate invasion, and recent studies have shown that antibody raised against the AMA1-RON2 peptide complex but not AMA1 alone protects against virulent *P*. *yoelii* infection in mice. In *Aotus* monkeys, AMA1-RON2 was superior to AMA1 alone as a vaccine to control blood stage growth of virulent *P*. *falciparum*.^29^ Compared to AMA1 alone, AMA1-RON2 increases serum GIA measured in vitro without increasing anti-AMA1 titers.^30^ The complex may enhance the immunogenicity of some conserved AMA1 epitopes. Preclinical studies are evaluating potential human-use adjuvants in rats as well as multi-allele AMA1-RON2 vaccines in order to induce significantly higher strain-transcending blocking activity.

## Transmission-blocking vaccines (TBVs)

In his opening remarks, Dr. Socrates Herrera from the Institute of Immunology in Cali, Colombia highlighted the increasing number of TBV studies, including discovery of novel antigens. He discussed the logistical, regulatory, and ethical challenges for establishing clinical trial sites in endemic areas, as well as the challenges to measure efficacy and evaluate immune responses against TBVs.

Dr. Patrick E. Duffy, chief of the NIAID Laboratory of Malaria Immunology and Vaccinology reminded the attendees of the seminal works of Carter and of Gwadz who showed that human immunity can act against sexual stage parasites in the mosquito,^31,32^ and presented results from clinical trials of two TBV vaccine candidates, Pfs25 (post-fertilization antigen) and Pfs230 (pre-fertilization antigen). To overcome intrinsically poor immunogenicity of the antigens, NIAID scientists conjugated both Pfs25 and Pfs230 to the immunogenic carrier protein ExoProtein (EPA) and administered them with adjuvants.^33^ Clinical trials of Pfs25-EPA formulated with Alhydrogel^®^ established that the antigen was safe and immunogenic in humans, and can induce functional antibodies that block parasite transmission to mosquitoes in laboratory assays.^34^ Furthermore, serum activity correlated with antibody titers. However, four doses were required to induce serum functional activity in most vaccinees, and functional activity was short-lived as anti-Pfs25 antibody levels waned rapidly after each dose. Notably, Pfs25 IgG titers dropped more rapidly than the titers against the carrier protein EPA. Ongoing trials are comparing and combining Pfs25 and Pfs230 vaccine antigens^34,35^ and assessing vaccine activity via standard membrane feeding assays (SMFAs) as well as by feeding mosquitoes directly on vaccinees (i.e., Direct Skin Feeding or DSF Assay). Pfs230 vaccine activity is known to be complement-dependent, and therefore fresh complement is required in the SMFA. In an ongoing trial, both Pfs25 and Pfs230 conjugate vaccines are being administered with AS01 adjuvant from GSK based on preclinical studies that this formulation might significantly increase antibody titer and therefore serum functional activity after vaccination.

Dr. Carolina Barillas-Mury, Chief of the Mosquito Immunity and Vector Competence Section from the Laboratory of Malaria and Vector Research at NIAID, reviewed new data that supports Pfs47 as a vaccine candidate. In studies from her group, Pfs47 appears to function as a “lock and key” to define compatibility between *P*. *falciparum* parasites and mosquitoes collected from different continents.^36^ Pfs47 proteins with compatible sequences allow the parasite to evade the *Anopheles gambiae* immune system, whereas parasites with incompatible sequences achieve only low infection rates. Pfs47 could have an important role in parasite transmission, and therefore might also be used as a target for intervention, as antibodies to a specific domain induce strong transmission-blocking activity as measured by SMFA. Ongoing studies from her group are investigating naturally acquired anti-Pfs47 antibodies in Malians, and the possibility that the Pvs47 orthologue could be modulating immune evasion in a *P. vivax* model.

Dr. Said Jongo, Head of Bagamoyo Clinical Trials Facility of the Ifakara Health Institute (IHI), Tanzania, highlighted Phase I to III activities at the IHI Clinical Trial facility, which opened in Bagamoyo in 2012 and has established itself as an important site for studies including malaria vaccine trials. IHI involvement in malaria research includes the assessment of safety and efficacy of novel antimalarial drugs and malaria vaccine candidates, the use of these modalities for malaria control and elimination, and the efficient translation of research results into policy improvement and community well-being. His team conducted the first CHMI in the modern era in Africa using PfSPZ injection, and conducted the first trial in Africa to assess the efficacy of PfSPZ-based malaria vaccine candidates using PfSPZ CHMI. This first experience with CHMI in Africa establishes its feasibility and can be a powerful platform for understanding malaria immunity and testing vaccines in the field.

## Basic aspects of vaccinology

One of the key limitations for the success of malaria vaccines is the difficulty in maintaining durable protection after immunization, which in part has been ascribed to poorly immunogenic antigens as well as to the effect of malaria itself in suppressing host responses. Efforts to increase the degree and durability of vaccine protection have included novel adjuvants and platforms as well as altered vaccine schedules, dosages and methods of delivery.^33,37–40^ However, long-lived protection has not been achieved. The session entitled “Basic Aspects of Vaccinology” brought together researchers interested in the factors that impair the host response to malaria as well as malaria vaccine efficacy.

Dr. Susan Pierce, from the NIAID Laboratory of Immunogenentics, emphasized that the immune response to malaria is complex. Despite repeated infections, sterilizing immunity is not achieved, while clinical immunity only develops after repeated infections and later in life. Many residents of malaria-endemic areas are asymptomatically infected.

Dr. Eleanor Riley, from the London School of Hygiene & Tropical Medicine, discussed the importance of understanding the immune responses induced by vaccines to aid future vaccine design and refinement. For example, combining data from phase 2 or phase 3 clinical trials with knowledge of the mechanism of action of vaccine-induced antibodies allows calculation of minimum effective antibody concentrations (MEC). Assessing the durability of these antibodies—specifically, the time period that concentrations remain above the MEC—could speed up the evaluation of new vaccine formulations. Worryingly, the MEC for anti-circumsporozite antibodies induced by RTS,S is very high (in the order of 1 mg/ml) and is maintained for only a few months after vaccination,^41^ and MECs for other invasion-blocking antibodies (e.g., anti-PfRH5 and anti-PfAMA-1) are of a similar order of magnitude. These data highlight the need to understand how to induce long-lived, high titer antibody responses that are needed to confer protection.

Dr. Peter Crompton, head of the Malaria Infection Biology and Immunity section at NIAID Laboratory of Immunogenetics, discussed factors beyond parasite antigenic variation and allelic diversity that may limit vaccine efficacy in endemic areas. Specifically, *Plasmodium* infections may subvert vaccine-induced immunity. In studies from Mali, malaria exposure is associated with atypical memory B cells that have decreased effector functions,^42^ Th1-polarized T follicular helper cells that exhibit impaired B cell help,^43^ and moncoytes that have a blunted inflammatory response.^44^ His studies support additional research to understand the impact of *Plasmodium* exposure on the quality of vaccine-induced responses, and to explore targeted interventions that could mitigate malaria-driven immunomodulation during vaccination.

Dr. John Harty from University of Iowa presented mechanisms underlying ineffective control of blood-stage *Plasmodium* infection. Using animal models, his team studied the importance of CD4 T cell responses in inducing protection against *Plasmodium*.

*P*. *yoelii* blood-stage infection in mice induces exhaustion of the CD4 T cell response. Consistent with this, in humans, *P*. *falciparum* infection induces higher expression of the inhibitory receptor PD-1 associated with T cell dysfunction.^45^ Previous studies have not established a consensus on the role of T regulatory cells (Treg) in the host response to *Plasmodium* infection, possibly due to variations in the parasite species, the animal model, and the model of infection. However, Tregs (Foxp3^+^ CD4^+^ T cell) expand in humans during malaria infection, and Tregs can potentially prevent parasite clearance by inducing a transient hiatus in the parasite-specific CD4 T cell responses. In mice, the effector CD4 T cell response to *P*. *yoelii* infection is biphasic and the hiatus coincided with Tregs that upregulated CTLA4. Blockade of CTLA-4 during the T cell hiatus leads to memory responses that confer species-transcending immunity to rechallenge.^46^ Notably, CTLA4 blockade before or after the CD4 T cell hiatus does not improve the CD4 T cell response or control of parasitemia. In summary, Tregs impede acute and long-term immunity against blood-stage malaria through CTLA4 expression, and this Treg activity manifests in a limited time window during the infection.

Dr. Dyann Wirth from the Harvard School of Public Health (Boston, USA) spoke on genotype-specific vaccine efficacy, initially noting that many *P*. *falciparum* antigens have extremely high diversity, incuding the major sporozoite surface antigen CSP (circumsporozoite protein) targeted by the RTS,S vaccine. Using Illumina sequencing data of parasite DNA from the RTS,S trials, the Harvard group tested whether the vaccine allele (3D7)-specific “sieve-effect” would result in fewer vaccine-match infections in vaccinated participants. The analysis indicated a greater cumulative efficacy against vaccine-match parasites, and also that 3D7 haplotype frequencies varied by study site throughout Africa.^47^ The results emphasize that parasite variation can limit the efficacy of malaria vaccines, and highlight the potential for parasite variants that evade vaccines to spread.

## Conclusions

Although there have been many efforts and substantial progress to control malaria, the disease is still a critical problem in endemic areas, affecting millions of children and adults. Vaccines aimed at different stages in the *Plasmodium* life cycle are in development and in the future, successful candidates could be combined to achieve the greatest activity. One candidate, the RTS,S vaccine that targets pre-erythocritic stage parasites, will soon be administered in a pilot implementation project in three African countries. In this report, we have highlighted recent advances by research groups involved in the arduous battle against malaria, reflecting the spectrum of activities from basic immunology through preclinical and clinical studies reported at the Malaria Vaccine Symposium. The question for malaria vaccines is no longer “if” but instead “when, for which purpose, and with what efficacy”.
